# MAGIC Database and Interfaces: An Integrated Package for Gene Discovery and Expression

**DOI:** 10.1002/cfg.399

**Published:** 2004-04

**Authors:** Marie-Michèle Cordonnier-Pratt, Chun Liang, Haiming Wang, Dmitri S. Kolychev, Feng Sun, Robert Freeman, Robert Sullivan, Lee H. Pratt

**Affiliations:** 1 Department of Plant Biology University of Georgia Athens GA 30602 USA; 2 Department of Systems Biology Harvard Medical School Boston MA 02115 USA

## Abstract

The rapidly increasing rate at which biological data is being produced requires a corresponding growth in relational databases and associated tools that can help laboratories contend with that data. With this need in mind, we describe here a Modular Approach to a Genomic, Integrated and Comprehensive (MAGIC) Database. This Oracle 9i database derives from an initial focus in our laboratory on gene discovery via production and analysis of expressed sequence tags (ESTs), and subsequently on gene expression as assessed by both EST clustering and microarrays. The MAGIC Gene Discovery portion of the database focuses on information derived from DNA sequences and on its biological relevance. In addition to MAGIC SEQ-LIMS, which is designed to support activities in the laboratory, it contains several additional subschemas. The latter include MAGIC Admin for database administration, MAGIC Sequence for sequence processing as well as sequence and clone attributes, MAGIC Cluster for the results of EST clustering, MAGIC Polymorphism in support of microsatellite and single-nucleotide-polymorphism discovery, and MAGIC Annotation for electronic annotation by BLAST and BLAT. The MAGIC Microarray portion is a MIAME-compliant database with two components at present. These are MAGIC Array-LIMS, which makes possible remote entry of all information into the database, and MAGIC Array Analysis, which provides data mining and visualization. Because all aspects of interaction with the MAGIC Database are via a web browser, it is ideally suited not only for individual research laboratories but also for core facilities that serve clients at any distance.


**Comparative and Functional Genomics**
*Comp Funct Genom* 2004; **5**: 268–275.
Published online in Wiley InterScience (www.interscience.wiley.com). **DOI:** 10.1002/cfg.399

**Conference Review**

### MAGIC Database and interfaces: an integrated package for gene discovery and expression

Marie-Michèle Cordonnier-Pratt[1]*, Chun Liang[1], Haiming Wang[1], Dmitri S. Kolychev[1], Feng Sun[1], Robert Freeman[2], Robert Sullivan[1] and Lee H. Pratt[1]*
[1] *Department of Plant Biology, University of Georgia, Athens, GA 30602, USA*
[2] *Department of Systems Biology, Harvard Medical School, Boston, MA 02115, USA*

**Correspondence to: Marie-Michèle Cordonnier-Pratt or Lee H. Pratt, Department of Plant Biology, University of Georgia, Athens, GA, 30602, USA. E-mail: mmpratt@uga.edu or leepratt@uga.edu*

### Abstract

**The rapidly increasing rate at which biological data is being produced requires a corresponding growth in relational databases and associated tools that can help laboratories contend with that data. With this need in mind, we describe here a Modular Approach to a Genomic, Integrated and Comprehensive (MAGIC) Database. This Oracle 9i database derives from an initial focus in our laboratory on gene discovery via production and analysis of expressed sequence tags (ESTs), and subsequently on gene expression as assessed by both EST clustering and microarrays. The MAGIC Gene Discovery portion of the database focuses on information derived from DNA sequences and on its biological relevance. In addition to MAGIC SEQ-LIMS, which is designed to support activities in the laboratory, it contains several additional subschemas. The latter include MAGIC Admin for database administration, MAGIC Sequence for sequence processing as well as sequence and clone attributes, MAGIC Cluster for the results of EST clustering, MAGIC Polymorphism in support of microsatellite and single-nucleotide-polymorphism discovery, and MAGIC Annotation for electronic annotation by BLAST and BLAT. The MAGIC Microarray portion is a MIAME-compliant database with two components at present. These are MAGIC Array-LIMS, which makes possible remote entry of all information into the database, and MAGIC Array Analysis, which provides data mining and visualization. Because all aspects of interaction with the MAGIC Database are via a web browser, it is ideally suited not only for individual research laboratories but also for core facilities that serve clients at any distance. Copyright © 2004 John Wiley & Sons, Ltd.**

Received: 6 February 2004
Accepted: 12 February 2004

**Keywords:** relational database; gene expression; microarray; electronic northern; electronic annotation; gene discovery

### Introduction

High-throughput production of biological data is becoming not only easier but also less expensive. The increase in the rate of data production brings with it a concomitant increase in the need for databases that not only facilitate data production but also provide efficient means for storing, mining and visualizing the data. As a 'single-investigator' research laboratory that began high-throughput sequencing in 1998, we faced these requirements in the absence of available, off-the-shelf software packages. The outcome has been the development of a database and associated tools that would meet not only our needs but those of other research groups as well. Given our initial interest in DNA sequencing, especially for production of ESTs, and subsequently in microarray applications, the

Copyright © 2004 John Wiley & Sons, Ltd.

software package that we developed focuses on issues related to gene discovery and gene expression.

The core of this package is a Modular Approach to a Genomic, Integrated and Comprehensive (MAGIC) Database, implemented in Oracle 9i. As the name implies, it is modular, such that new functionalities can readily be added, and both integrated and comprehensive, so that a user can move quickly among different kinds of information. The system is: object-orientated, to facilitate adding modules; generic, so that it will perform well in a wide variety of environments; and scalable. In addition, it offers a high degree of security, to facilitate mingling of public with private data and to ensure that access to data is available only to those given permission to view it. The package can also be used effectively on relatively inexpensive desktop computers, e.g. we have duplicated the entire system on one single-processor desktop computer with a Linux operating system. We summarize here the fundamental characteristics of the MAGIC Database and its associated interfaces.

### MAGIC database

The MAGIC Database currently serves several research programs, including public EST projects (sorghum, pine, horse, human embryonic stem cells, and the parasitic ciliate *Ichthyophthirius*) and a limited amount of BAC sequencing (rice). Data from these projects can be viewed at **http://fungen.org**. The database is designed, however, to also work with data coming from external sources. For example, 56 083 chromatograms from the Sederoff pine EST project (**http://pine.ccgb.umn.edu/**) have been processed into the database and can be explored at **fungen.org**, while 65 190 chromatograms produced by the Whitehead Institute (**http://www.broad.mit.edu/**) have similarly been processed into the database. In the latter case, the investigators providing the chromatograms are working directly with our production database, using the tools described below. The microarray segment of the MAGIC Database is more recent, but at this writing already contains information associated with 89 microarray experiments, using arrays consisting of 17 732 features. All data have been entered from a remote location, and data analysis and visualization are also performed via the Internet. The result is an integrated database that combines the gene discovery capabilities from high-throughput DNA sequencing with the gene expression data derived from microarray experiments. While the two segments of the database will be described independently below, a major strength remains the tight integration that facilitates mining all information simultaneously.

### MAGIC gene discovery

MAGIC Gene Discovery can be dissected into numerous interlinked subschemas or modules. One of these is MAGIC Admin, which not only provides administrative support but also controls processing of sequence chromatograms. MAGIC SEQ-LIMS consists of tables that track sequencing activity in the laboratory, yielding the chromatograms to be processed. Processing pipelines, written in Perl and using common bioinformatics tools, enter sequence data into tables in MAGIC Sequence, while information about clustering of these sequences, together with the results of such analyses, is entered into MAGIC Cluster. MAGIC Annotation holds electronic annotations of both cluster consensus sequences and individual ESTs obtained by high-throughput BLAST (Altschul *et al.*, 1990, 1997) and BLAT (Kent, 2002). In addition, pipelines are in place for identifying and classifying microsatellites (SSRs) and single nucleotide polymorphisms (SNPs). The output of these pipelines is entered into MAGIC Polymorphism. An additional subschema, MAGIC Map, is currently in development. It will provide support for the display of sequence and gene expression information in the context of a genetic or physical map. This portion of the MAGIC Database can be explored further at **http://fungen.org/Projects/Sorghum/PAG2004.pdf**.

Interfaces for updating tables, performing queries and visualizing the results of those queries are all either Java Server Pages (JSP) or Java GUIs deployed through Java Web Start. These interfaces include MAGIC Administration, which facilitates the creation and management of laboratory groups, user accounts and access controls, as well as the definition of sequencing projects, including vector/primer/adapter information required for processing chromatograms. Other interfaces provide data tracking and quality control information, as well as access to SSRs and SNPs. Additional

Copyright © 2004 John Wiley & Sons, Ltd. 

information about these interfaces can be found at **http://fungen.org/Laboratory/Bioinformatics.htm**. Two additional interfaces, which will be described here, are MAGIC Sequence Viewer and MAGIC Gene Discovery, both Java GUIs that are launched by Java Web Start.

### MAGIC sequence viewer

Upon logging in, a user may select the library, vector or EST direction and, if desired, data deriving from a specific 96-well plate or block (Figure 1, upper left). Sequences are listed in a second window in tabular format, where each row includes a unique name following a strict convention defined by us (Figure 1, upper right). This tabular display includes information about EST direction, presence of vector at the beginning (VF1) or end (VF2) of a sequence, length of any PolyT tail, length of a sequence as trimmed of vector, adaptor and low quality ends (Q16VS), and whether a sequence is entirely vector (TotV). There are also columns for an alias name, which is a unique identifier to accommodate a second naming convention, and an AKA name (not shown), which need not be unique. Rows can be sorted by values in any column by clicking on the appropriate column header. A FASTA file of one or more selected sequences (trimmed and reverse complemented as desired) can be created and downloaded from this window. In addition, a failure report that quantifies all factors contributing to sequence failures can be initiated from this interface.

When a sequence is selected by clicking on a row, it is displayed with both text and graphical views (Figure 1, bottom). The latter reveals phred quality scores as a function of sequence position (Ewing and Green, 1998). Sequences can be filtered by any one of several options, with the selection being highlighted in both views. The Q16VS100 option shown here identifies that part of a sequence that is free of vector, adaptor, polyT and contaminating sequence such as *E. coli* and ribosomal DNA, that has been trimmed of low quality regions identified by a sliding-window and gap-joining algorithm using a phred score of 16 as the cut-off, and that remains at least 100 nt in length. A Q16VS100 sequence is one of sufficient quality that it can be submitted to GenBank. A region can also be selected with a mouse, and, if it consists of a single nt, then one can identify individual phred scores. A BLAST function submits whatever region has been selected either to NCBI or to a target database at **http://fungen.org/blast.html**

### MAGIC gene discovery

This Java GUI opens with a window that permits a user to select the results of a specific clustering analysis. Once selected, a second window provides multiple options for querying the database and viewing data returned (Figure 2, bottom). One can examine clusters and rate of gene discovery (Cluster Overview), search for clusters satisfying selected query parameters (Search Contig and Search Subgroup), search for sequences that meet selected query parameters (Search Annotation), identify full-coding-length clones (Search Full Length Seqs), or explore the results of *ad hoc* high-throughput BLASTs (Standalone Blast Viewer). Figure 2 (bottom) displays the default view after processing and downloading selected data. Each vertical bar represents a cluster; colour-coded segments represent different libraries and the height of each segment is proportional to the count of ESTs per library for that cluster. Thus, finding a gene that is preferentially expressed in a given cDNA library can be as simple as searching for a cluster that is predominantly or exclusively the desired colour. A comparable search can be done in high-throughput fashion at the Search Contig or Search Subgroup pages (see below).

Clicking on a cluster of interest opens a pie chart displaying EST distribution among the different libraries (Figure 2, upper left). Selecting the 'Expression by Subgroup' option in this first chart opens a second that reveals an aggregate expression profile for user-defined groups of libraries (Figure 2, upper right). Selecting 'Group and Library Definition' or 'Subgroup Definition' in one of these two windows provides the desired information in a fourth window (Figure 2, upper middle). The first chart also provides direct access to electronic annotations, which are also available from the Search Annotation page, and to the results of a BLAT analysis. At present, the latter displays alignments of all possible pairwise BLAT sequence comparisons within the cluster. BLAT to genome sequence is presently being added. The 'Sequences in Contig and Consensus' option permits a user to download all data in FASTA

Copyright © 2004 John Wiley & Sons, Ltd. 

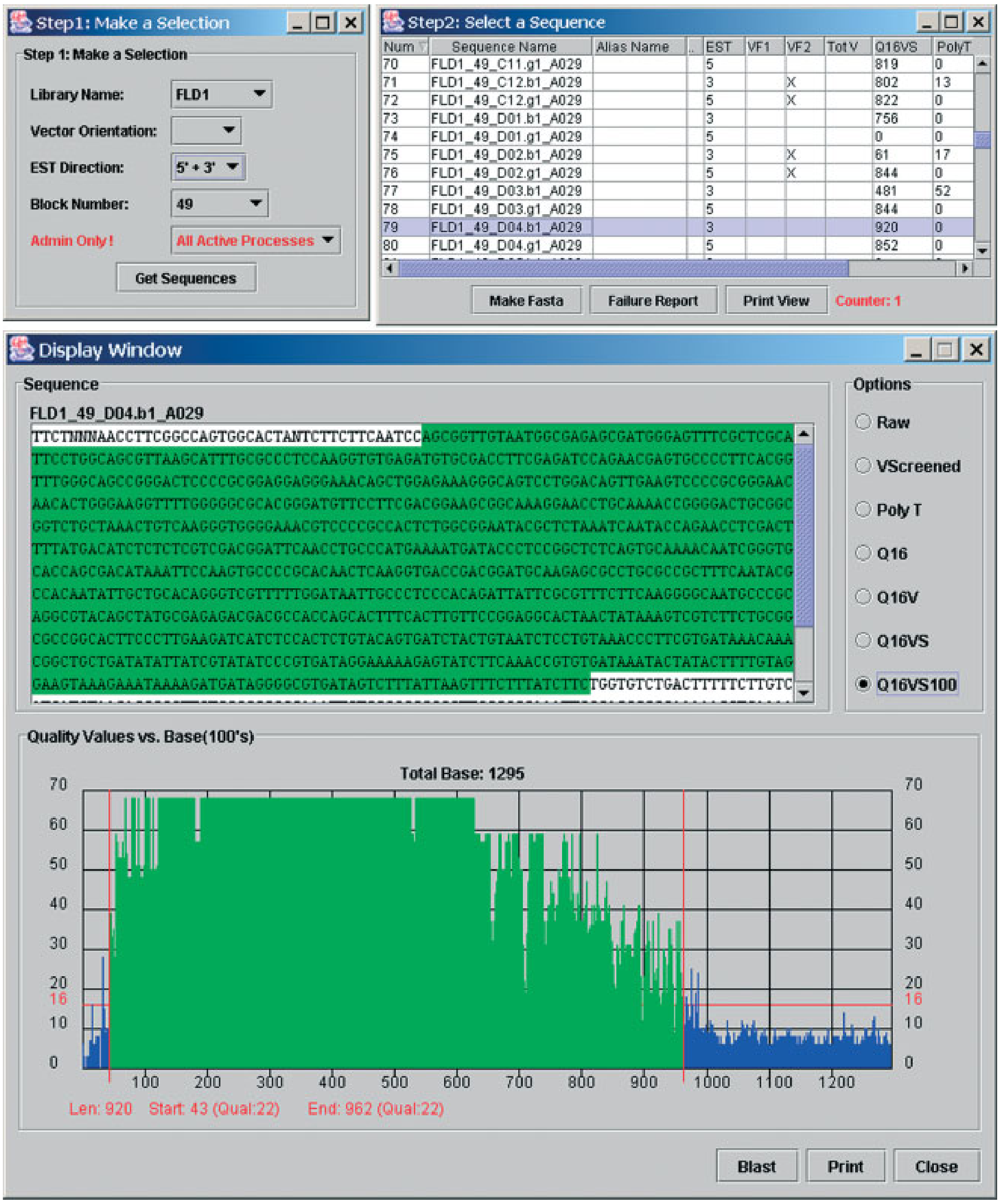

**Figure 1.** MAGIC Sequence Viewer. After a successful login, the user selects a library, vector orientation or EST direction and, if desired, a specific block number for display (upper left). Clicking on 'Get Sequences' lists all selected sequences in a new window (upper right). Selecting a row displays the specified sequence in the Display Window (bottom). The upper and lower parts of this window display a selected sequence in text and graphic formats, respectively. In the latter, phred quality scores are plotted as a function of position. The sequence as shown is highlighted to reflect the part that is of sufficient quality to submit to GenBank. Additional details are provided in the text format and also provides a direct link to NCBI BLAST. 'Contig Alignment' displays sequence alignments, colour-coded to reveal discrepancies from the consensus sequence and to identify base calls with low phred scores. Sequences in this alignment can be sorted by offset from the consensus, by sequence name, or by the genotype from which each sequence was derived. The latter is

Copyright © 2004 John Wiley & Sons, Ltd. 

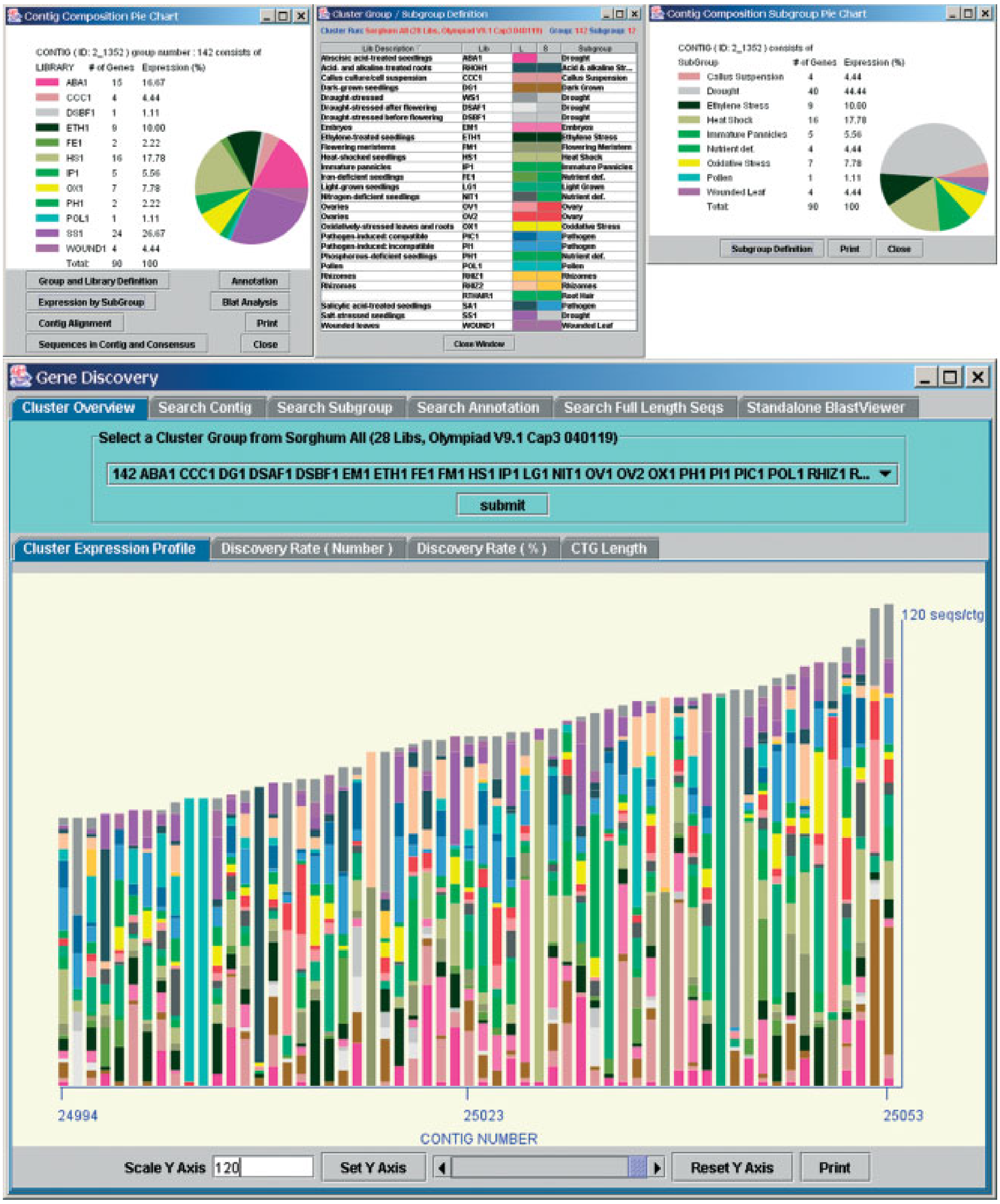

**Figure 2.** Cluster expression profiles as viewed in MAGIC Gene Discovery. Following selection of a cluster group, the EST distribution within individual clusters is displayed (bottom). Each vertical bar and its coloured segments represent the EST count per library per cluster. Clicking on a bar displays a new chart window (upper left) containing more detailed information about the constituent cDNA libraries. Clicking on 'Expression by Subgroup' displays similar information for user-defined groups of libraries. Selecting 'Group and Library Definition' or 'Subgroup Definition' presents the requested information (upper, middle)

especially useful for visual identification of SNPs and haplotypes, although a high-throughput search is available in an interface that can be selected at **http://fungen.org/Sorghum.htm**

The 'Search Annotation' option offers tabular and visual displays for BLAST data of all sequences to a variety of databases and offers annotation based on the best BLAST return from

Copyright © 2004 John Wiley & Sons, Ltd.

high-throughput BLAST to the PIR-NREF database (Huang *et al.*, 2003). From the tabular display, a user can select or order by sequence name, cluster ID, HSP, Score, Expect value, percentage identity, percentage positive, offset, NREF ID, target protein name, target species, target protein length, target protein function, first and last nucleotide of the query sequence in the match, first and last amino acid of the target sequence in the match, and the reading frame of the query sequence in the match. Selecting a row reveals the match in text format and provides a link back to the appropriate entry in the PIR database. Any combination of selected rows may be exported to a subtable that can be queried in similar fashion to the first. This process can be repeated indefinitely. Thus, one can quickly identify subgroups of data that meet several selected criteria. This interface provides a biologist with a simple but effective substitute for what would otherwise require a complex SQL query.

Of greatest interest to a biologist, perhaps, is the ability within the Search Contig or Search Subgroup pages to request all clusters that contain, for example, six or more ESTs where at least 70% of the cluster members derive from a selected library or subgroup of libraries. Results returned in response to queries on these and other pages are presented in tabular form, with the ability to sort by any column and to create subtables iteratively. Selecting any sequence or cluster, depending upon the page, will reveal the alignment of the best BLAST return from PIR-NREF. Moreover, all of the information presented on the Search Annotation page is also provided on these pages, together with direct links to a pie chart for a selected cluster, and to the other options available from the chart window (Figure 2).

### MAGIC microarray

Tables dedicated to microarray applications store user information, results of sample tracking during cherry picking and array construction, genotype information, general project information, biomaterial to RNA data, protocols, hybridization and analysis information, and materialized views utilized by Spotfire for data visualization and analysis. Via materialized views, Spotfire can directly access and visualize in real time any information in MAGIC that can be linked to a given microarray feature, including annotations. MAGIC Array Data Manager enables remote entry of data into the database. Thus, MAGIC Microarray constitutes a distributed microarray bioinformatic resource that can serve laboratories anywhere via the Internet, while simultaneously associating microarray features with other information deriving from MAGIC Gene Discovery.

### MAGIC array data manager

This Java GUI (Figure 3) facilitates remote entry of all laboratory information concerning a microarray into the MIAME-compliant (Brazma *et al.*, 2001) MAGIC Microarray section of the database. The look-and-feel of this interface is much like that of MADAM (Saeed *et al.*, 2003; **http://www.tigr.org/software/**), from which we began its development. Data entry via this interface begins with the definition of a project and all experimental protocols and moves through definitions of biomaterial, growth or culture conditions, experimental treatments, experimental designs, definition of experimental variables and specific experiments, to definition of experimental and biological replicates and finally RNA preparation (RNA page, not shown). A second section enumerates creation of array designs, preparation of labelled cDNA, definition of either one- or two-step hybridization protocols, execution of a specific hybridization, and finally scanning of an array to produce a scan pair of TIF files (Array page). Figure 3 displays the page that is used to enter a complete description of the RNA used to prepare a labelled cDNA probe, including the experimental and biological replicates from which it was derived (upper right).

This interface also assists an investigator in specifying the two TIF files associated with a given bar-coded array and uploading those files via sFTP to the database. After a successful upload, the same interface aids the user with spot detection and data extraction/deposition to a defined location. From this location the data are parsed into the database, from which they are later retrieved, formatted for submission to MIDAS and normalized (Saeed *et al.*, 2003; **http://www.tigr.org/software/**). The normalized data files are parsed and database tables updated.

After data deposition and analysis, users with the appropriate access permissions view the data from our Spotfire server via their licensed Spotfire browser. A decision tree guides the remote user in

Copyright © 2004 John Wiley & Sons, Ltd. 

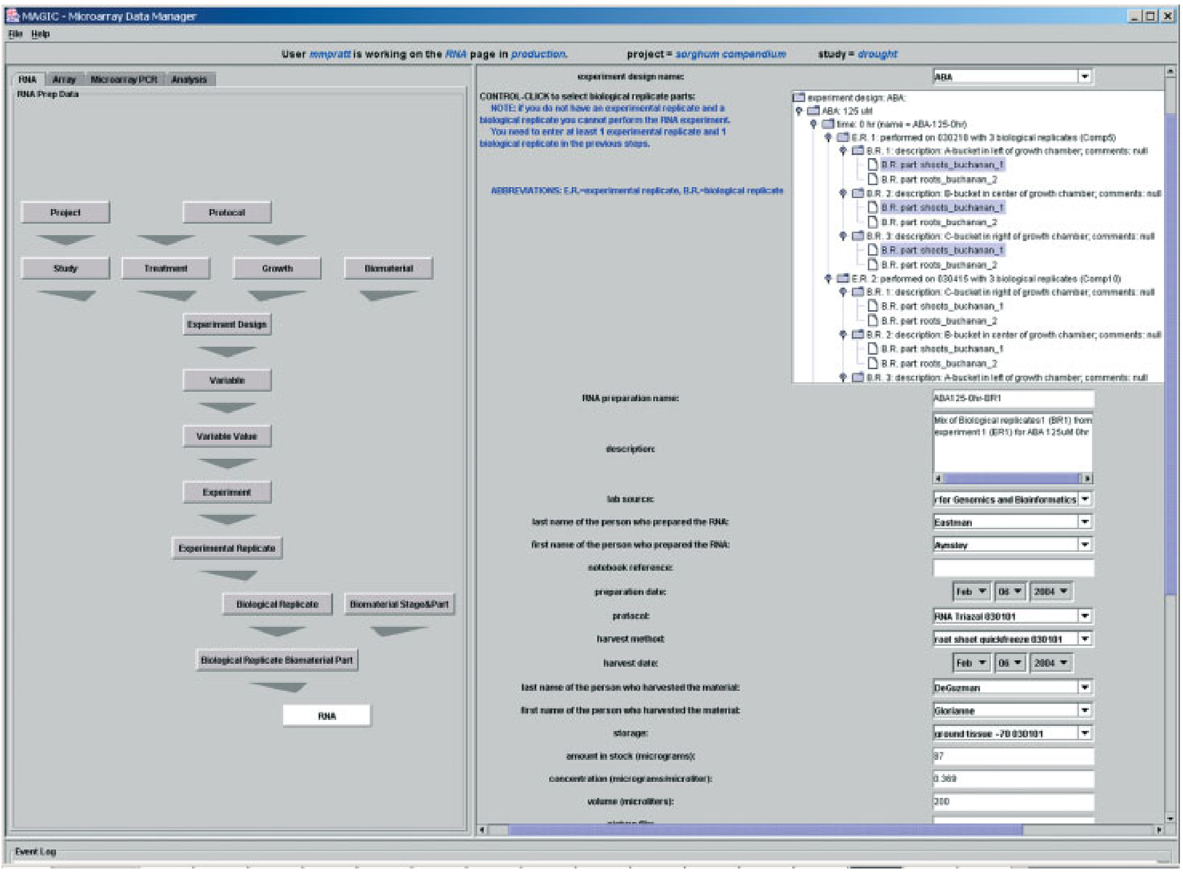

**Figure 3.** The MAGIC Array Data Manager facilitates entry of data into the MIAME-compliant MAGIC Microarray section of the MAGIC Database. The RNA page shown here illustrates the extensive level of detail handled by this interface finding desired data, which are then downloaded together with annotations and other information from the MAGIC Gene Discovery portion of the MAGIC Database. This dataset can be retained locally. Spotfire facilitates creation of typical visualizations used with microarray data, including scatter plots and heat maps. Numerous filtering options enable a user to select with great precision the data to be displayed, while a 'Details-on-Demand' window displays all relevant information about selected spots retrieved from the MAGIC Database. Spotfire also provides additional analytical capabilities, including hierarchical clustering, K-means clustering, self-organizing maps, principal component analysis and numerous statistical functions. Documented APIs permit extensive customization. As an example of these capabilities, we created a Spotfire Web Link to determine quickly whether microarray results are consistent with digital expression profiles obtained from ESTs in the Gene Discovery Contig view. With a few simple steps, a user can tentatively validate microarray results without leaving the computer, leaving more definitive validation to a later time.

### Availability

Both the MAGIC Sequence Viewer and MAGIC Gene Discovery tools can be explored at **http://fungen.org/seqview/** and **http://fungen.org/genediscovery/**, respectively. At these sites, the versions of these Java GUIs are at least one version behind what is used for production. Consequently, all features described here might not yet be available. Other interfaces used for internal processes are of little or no value to the public and thus are not available there. However, additional information about them can be obtained at **http://fungen.org/Laboratory/Bioinformatics.**

Copyright © 2004 John Wiley & Sons, Ltd. 

**htm** and **http://fungen.org/Projects/Sorghum/PAG2004.pdf.** We intend to make all code available as open source when development has reached an initial stage of completion in early 2005. Prior to that full release, the authors welcome requests to obtain the Oracle DDL script for database installation as well as executable code for the interfaces, with the understanding that support can be provided on only a limited basis prior to public release.

### Acknowledgements

We thank John Quackenbush, Jianwei Li and Joseph White at The Institute for Genomic Research (TIGR) for advice and helpful discussions. This research was supported by grants from the National Science Foundation Plant Genome Research Program (DBI-0111040 to M-MC-P and LHP, DBI-0115911 to Jeffrey F. D. Dean, University of Georgia, and 02100807 to Peggy Ozias-Akins, University of Georgia).

### References

Altschul SF, Gish W, Miller W, Myers EW, Lipman DJ. 1990. Basic local alignment tool. *J Mol Biol* **215**: 403–410.

Altschul SF, Madden TL, Schaffer AA, *et al*. 1997. Gapped BLAST and PSI-BLAST: a new generation of protein database search programs. *Nucleic Acids Res* **25**: 3389–3402.

Brazma A, Hingamp P, Quackenbush J, *et al*. 2001. Minimum information about a microarray experiment (MIAME) — toward standards for microarray data. *Nature Genet* **29**: 365–371.

Ewing B, Green P. 1998. Base-calling of automated sequencer traces using *Phred*. II. Error probabilities. *Genome Res* **8**: 186–194.

Huang H, Barker WC, Chen Y, Wu CH. 2003. iProClass: an integrated database of protein family classification, function and structure information. *Nucleic Acids Res* **31**: 390–392.

Kent WJ. 2002. BLAT — the BLAST-like alignment tool. *Genome Res* **12**: 656–664.

Saeed AI, Sharov J, White J, *et al*. 2003. TM4: a free, open-source system for microarray data management and analysis. *Biotechniques* **34**: 374–378.

Copyright © 2004 John Wiley & Sons, Ltd.

## References

[PDF_00457] Altschul S. F., Gish W., Miller W., Myers E. W., Lipman D. J. (1990). Basic local alignment search tool.. J Mol Biol.

[PDF_00459] Altschul S. F., Madden T. L., Schäffer A. A., Zhang J., Zhang Z., Miller W., Lipman D. J. (1997). Gapped BLAST and PSI-BLAST: a new generation of protein database search programs.. Nucleic Acids Res.

[PDF_00462] Brazma A., Hingamp P., Quackenbush J., Sherlock G., Spellman P., Stoeckert C., Aach J., Ansorge W., Ball C. A., Causton H. C. (2001). Minimum information about a microarray experiment (MIAME)-toward standards for microarray data.. Nat Genet.

[PDF_00465] Ewing B., Green P. (1998). Base-calling of automated sequencer traces using phred. II. Error probabilities.. Genome Res.

[PDF_00468] Huang Hongzhan, Barker Winona C., Chen Yongxing, Wu Cathy H. (2003). iProClass: an integrated database of protein family, function and structure information.. Nucleic Acids Res.

[PDF_00471] Kent W. James (2002). BLAT--the BLAST-like alignment tool.. Genome Res.

[PDF_00473] Saeed A. I., Sharov V., White J., Li J., Liang W., Bhagabati N., Braisted J., Klapa M., Currier T., Thiagarajan M. (2003). TM4: a free, open-source system for microarray data management and analysis.. Biotechniques.

